# Membrane of Egg for Skin Grafting

**Published:** 1884-11

**Authors:** 


### Membrane of Egg for Skin Grafting.

In a case of extensive burn unhealed after six years, Dr. Frank C. Wilson, of Louisville, Ky., in *Med. News*, says: "I made use of three different kinds of skin grafts, namely, from the skin of a young rabbit, from the human skin, and from the inner membrane of a perfectly fresh hen's egg." Of the three he much preferred the egg membrane as being much more readily obtained, and one egg will supply any number of grafts needed.

First Party: "Have you any court-plaster?" Second Party (a druggist): "No, but here's some sticking-plaster. You see we have no royal family in this country, and therefore no court, and consequently no court-plaster. If your finger is cut you will have to take a piece of the democratic article, or go somewhere else."

Rules in Treating Children.—A medical writer has wisely said: "One of the watchwords in treating children is elimination. Do not lock up the secretions. Give nature, that grand old mother, a chance. Very rarely should opium or any of its preparations, or derivatives, be used in the treatment of children.

Latin is a "dead language"—when an inexperienced drug clerk fools with it.

The International Medical Congress of 1884 may be written down as a great success; 1,600 medical men attended it, 1,200 of whom came from foreign countries. Some of the work done will leave a lasting impress on science; the reception will leave an impress for ever upon those who were fortunate enough to share in it; so that the name of Denmark will become a cherished memory.

VIRCHOW ON TRICHINOSIS.—Prof. Virchow has written to the newspapers to express his opionion that the danger arising from trichinosis in German pork is "infinitely greater" than the peril of an epidemic from the American hog, and that, to be consistent, the Imperial Goverment, which has forbidden the importation of all sorts of American pork, should not allow swine-rearing in Germany.

The word "microbe," now so commonly used, was coined by M. Charles Sedillot, of Strasbourg, in February, 1878, in a paper which he read on the application of M. Pasteur's discoveries to surgery. Coming from the Greek words *mikros*, small, and *bios*, life, it aptly describes the thing intended. In replying to M. Sedillot, M. Pasteur used the word twice, and scientific men have since generally adopted it.

MENTHOL.—I think it was Mr. Malcolm Morris, who, some time ago, spoke of the antiseptic use of menthol in ringworm of the scalp. I applied a solution in rectified spirit to the tinea tonsurans of a young friend in the winter of 1879, and cure resulted; and recently I have met with success in this disease from a pomade of menthol, iodoform, and vaseline.

In facial neuralgia, in some forms of sciatica, in neuralgic headache (clavus), and in toothache—the most recent instance being in a severe case of a lady recovering from acute alcoholism—I have repeatedly found relief to follow within a few minutes of its application. The cones or sticks are useful for external application. Frequently, however, I use now the following formula: Menthol, 30 grains; and spirit of rosemary, rectified spirit, each two drachms; or compound spirit of lavender may be used instead of the rosemary for application to the cavity of a carious tooth. I have only taken it once internally myself, but never have prescribed it. This has, however, been done.—*Archibald D. Macdonald, M. B., Edin., in British Medical Journal.*

BUDDHISM AND THE TRANSMISSION OF DISEASES.—The Buddhist law is significantly stringent on this point—so much so that, according to Jardine's translations of the Burman's law of "Marriage and Divorce," we observe that, should man or woman unwittingly marry into a family afflicted with certain hereditary dieseases, as leprosy, cancer, syphilis, madness, etc., which in the Dhammathat relating to marriage are collectively designated "*hinaroga*, or wasting diseases," he or she may sue out a divorce. Buddhists are taught to believe that not only will the children of diseased parents inherit a transmissible disease, but that the husband or wife will contract the same by association—living together under the same roof.

AURI ET SODII CHLORIDUM.—The pure salt is composed of equal parts of chloride of gold and chloride of sodium. It is soluable in water, it promotes glandular secretions, and the elimination of waste products by increasing the capillary and arterial circulation. Its administration is indicated in disease produced and characterized by inactivity of function, due to a retarded or enfeebled circulation in the organ, a valuable remedy then in indigestion and constipation. It is used with benefit in headache and melancholia, when due to disturbances in digestion and elimination. When the functions of the brain are depressed, the mental activity diminished from the continuous use of alcohol and narcotics, the chloride of gold and sodium is administered with benefit.

